# The Effects of Non-Nutritive Sweetener Consumption in the Pediatric Populations: What We Know, What We Don’t, and What We Need to Learn

**DOI:** 10.3389/fendo.2021.625415

**Published:** 2021-04-01

**Authors:** Betty Shum, Senta Georgia

**Affiliations:** ^1^ Center for Endocrinology, Diabetes, and Metabolism, Department of Pediatrics, The Saban Research Institute, Childrens Hospital, Los Angeles, CA, United States; ^2^ Department of Stem Cell Biology & Regenerative Medicine, Keck School of Medicine, University of Southern California, Los Angeles, CA, United States

**Keywords:** pediatrics, microbiome, non-nutritive artificial sweeteners, metabolic disease, diabetes

## Abstract

Childhood obesity is increasing at an alarming rate in the United States. This trend carries serious risk of children developing obesity-related diseases including Type 2 diabetes and cardiovascular disease. Non-nutritive sweeteners (NNS) are used as substitution for table sugar as a way to prevent weight gain. Their consumption is ubiquitous in adults and children; however the long-term health outcomes of chronic NNS consumption in children are unclear. Conflicting observational studies suggest that children consuming NNS are at risk of obesity and development of type 2 diabetes, while others concluded some benefits in weight reduction. Here, we review the physiological mechanisms that can contribute to the negative metabolic effects of NNS. We will focus on how NNS alters the sweet perception leading to increase caloric consumption, how NNs alters the gut microbiota, and how NNS may disrupt glucose homeostasis and initiate a vicious cycle of pancreatic endocrine dysfunction. Studies focused on the pediatric population are limited but necessary to determine whether the potential weight loss benefits outweigh the potential negative metabolic outcomes during this critical development period.

**Graphical Abstract:**
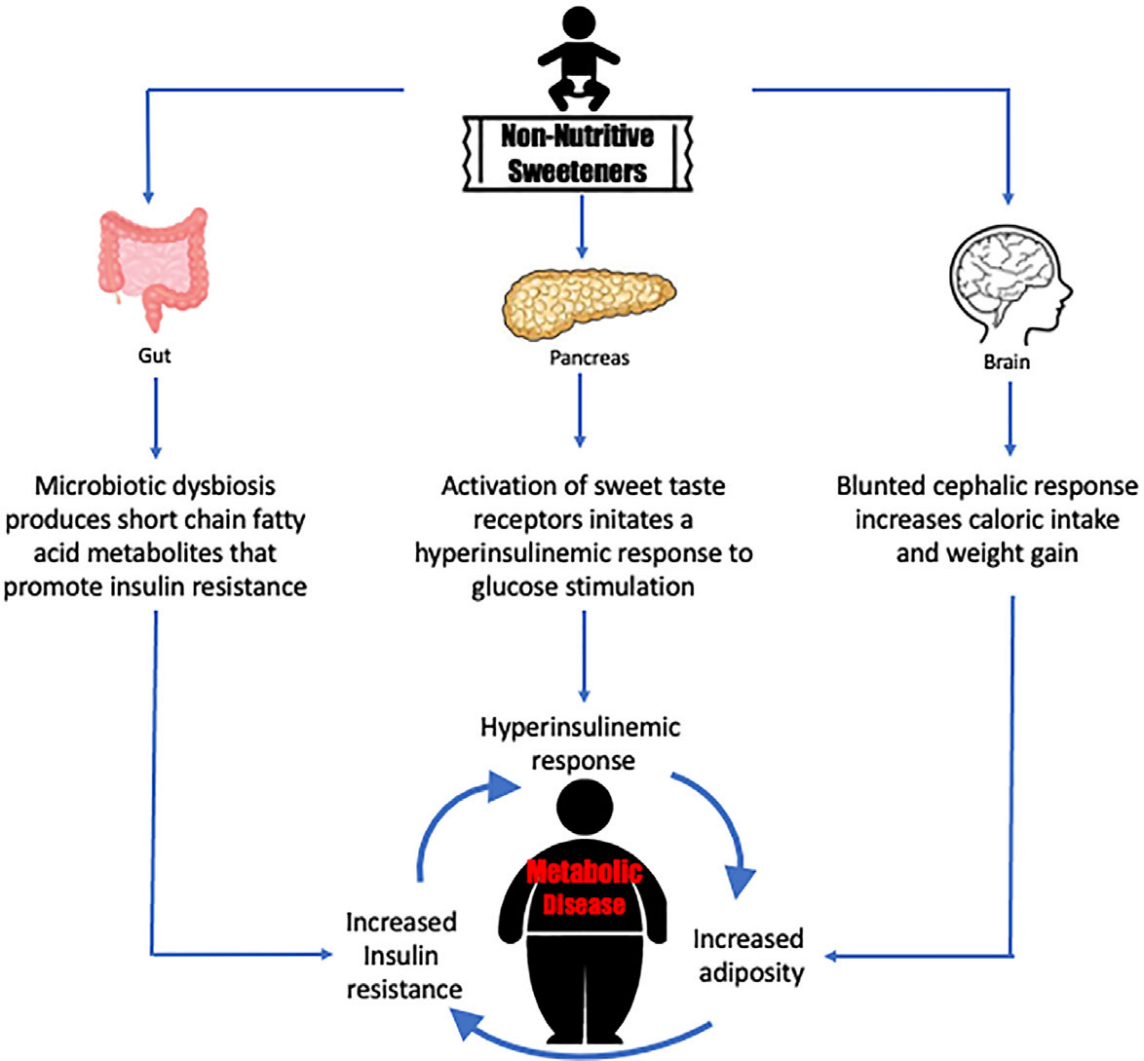
Non-nutritive sweeteners may drive a cycle of responses that precipitates metabolic disease.

## Highlights 

The market for non-nutritive sweeteners (NNS) is billion-dollar industry with growing demands from consumers for who want healthier foods that replace table sugar with a sweet-tasting, no-calorie alternatives. Children are a growing target market for this industry, but most parents are not aware that food products labeled as reduced sugar are substituted with NNS. As NNS are increasingly consumed by children, it becomes important to study the long-term safety and health outcomes of NNS consumption in children. As reviewed below, the data has not been conclusive for weight reduction benefits and some observational studies suggest the alternative of increasing risk for type 2 diabetes and cardiometabolic diseases in children. For scientists designing experiments to investigate the mechanistic pathways of NNS effects, it would be important to investigate how in-utero exposure and early childhood exposure to NNS influences metabolic outcomes in adulthood. As clinicians counsel patients and parents about NNS use, it is important to be aware that the competing interests of the food industry and the long-term health outcomes of NNS consumption may not align.

## Introduction

Childhood obesity is one of the most serious public health concerns, affecting 1 in 5 children and adolescents in the United States (1). This alarming rise in prevalence has significant health outcomes; childhood obesity is linked to the development of Type 2 diabetes, cardiovascular disease, and metabolic syndrome (2–4). Studies have found a strong positive association between the consumption of added sugars and the risk of developing obesity and type 2 diabetes (5–7). To help with preventing weight gain and glucose excursion, the use of non-nutritive sweeteners (NNS) has been used as an alternative to caloric sweeteners in beverages and food products. However, it is not clear if the benefits of weight reduction outweigh the potential negative health outcomes including type 2 diabetes, nonalcoholic fatty liver disease, and metabolic syndrome (8–10). Despite the fact that NNS consumption has become ubiquitous among children and adolescents, very few studies that have looked at the health outcomes of NNS in the pediatric population (11, 12).

Six NNS are approved for use by The Food and Drug Administration (FDA), including aspartame, acesulfame-potassium, neotame, saccharin, sucralose, and advantame. Two natural sweeteners, stevia and monk fruit, have been approved as generally recognized as safe (GRAS). NNS that were once considered inert, like saccharin, have been reported to have negative metabolic effects in observational studies, including increasing weight gain, adiposity, and risk of developing T2DM (8–10, 13). The underlying biological mechanisms that drive these metabolic effects remain unknown and represent a critical gap in knowledge that needs to be filled, as this may change clinicians recommendations for or against NNS use in the pediatric populations.

This review will discuss the physiological mechanisms that contribute to the negative metabolic effects of non-nutritive sweeteners and will identify research opportunities that could advance our understanding of their effects on the pediatric population. We will focus on how NNS alters the sweet perception leading to increase caloric consumption, how NNS alters the gut microbiota, and how disrupting metabolism and glucose homeostasis can cause pancreatic endocrine dysfunction.

## NNS, Sweetness Perception, and Caloric Consumption

NNS are used as a substitute for sugar because its perceived taste can be up to 20,000 times sweeter. Sugar consumption activates sweet taste receptors found on the tongue (14). This signals to the brain that calories are expected and initiates the cephalic phase response. The cephalic phase response is a cascade of anticipatory hormonal signals that increase insulin secretion, gastric enzyme production, and initiates satiety signaling (15). NNS activate the same sweet taste receptors and initiates the cephalic phase response (14); however, the normal physiological response is disrupted by the mismatch in caloric energy intake (16). The anticipatory Pavlovian conditioning of the cephalic response is never satisfied by NNS consumption since the sweet taste stimulus is not followed by caloric intake, thus leading to an impaired balance of energy intake and sweet signal activation.

To test the hypothesis that NNS disrupts the physiological balance between sweet taste and caloric content of food, Swithers and Davidson fed male Sprague-Dawley rats either saccharin-sweetened or glucose-sweetened yogurt (16). They found that rats fed saccharin-sweetened yogurt had higher weight gain, greater adiposity, and increased total energy intake compared to rats fed glucose-sweetened yogurt. Glucose-exposed rats had decreased calorie intake after a high caloric pre-meal while the saccharin-exposed group had no change in calorie intake with a pre-meal. These findings suggested that NNS-mediated mismatch of the cephalic phase response and caloric consumption led to metabolic dysfunction.

Swithers and colleagues later compared the effects of acesulfame-K on calorie intake and weight gain (17). Rats that consumed acesulfame-K demonstrated increased energy intake and body weight gain when compared to glucose-consuming control group. The study also investigated whether the mode of delivering the sweeteners affected the metabolic outcomes. They found that rats consuming saccharin-sweetened refried beans had higher weight gain compared to rats that consumed saccharin-sweetened yogurt (17). In contrast to Swithers studies, Palmnas and colleagues reported that chronic consumption of low dose aspartame in the water of a diet-induced obese rat model resulted in a lower body mass than those that consumed sugar-sweetened water (18). The aspartame-exposed groups consumed fewer net calories but were found to have fasting hyperglycemia and impaired insulin tolerance. The inconsistent findings of NNS effects on weight gain raise questions as to what are the underlying mechanisms that explain these outcomes.

Few studies have investigated the metabolic outcomes in children that consume NNS. The limited observational studies of pediatric patients have suggested a positive association between increased BMI in children that consume NNS-sweetened carbonated soft drinks (19–21), weight gain (22), increased body fat accumulation (23–26), and obesity (27). However, because these studies were mostly observational, they could not conclude that there is a direct causality of NNS consumption with weight gain. In contrast, randomized control trials suggest there is reduced weight gain when regular soda is replaced with NNS soda in children and adolescents (28–30).

Because NNS are not adding calories to the diet to directly drive weight gain, it is possible that a blunted cephalic phase response may play a critical role in this phenomenon. Children who consume NNS beverages were found to have higher caloric and carbohydrate intake compared to water only consumers (31). This observation could be explained by the dysregulation of the predictive relationship between sweetness perception and caloric intake leading to a positive energy balance. Studies have revealed of children with early exposure to sugar sweetened foods have a higher preference of sweet taste and food high in sugar (32). However, it is unclear whether early exposure to NNS also leads to intense preference for sweet taste. Functional brain MRI studies performed in healthy adults have shown different parts of the brain is activated in response to NNS compared to regular sugar, suggesting that NNS may alter the taste and reward pathway system (33, 34). Studies in children that investigate if exposure to NNS changes in brain response to sweeteners should be initiated.

It is critical to investigate if the proposed physiological mechanisms underlying NNS effects on weight gain and adiposity have a long-term consequences in pediatric patients. Early exposure to NNS in childhood may alter the adaptive physiological responses in the reward pathway and sweet taste preference, thus influencing the eating pattern from childhood into adulthood. Additionally, future studies should be designed to investigate dysregulation of the predictive relationship between sweet perception and calorie ingestion in children. It is critical to understand the longitudinal effects of NNS exposure starting in early childhood through adolescence and into adulthood to answer these pressing questions.

## NNS and Gut Microbiota

NNS have been shown to alter the gut microbiota in animal studies and a limited number of human studies, suggesting they may have secondary role in producing metabolic dysregulation (18, 35–39). Diet is a major factor in modulating the gut environment and shifts in the gut microbiota populations may influence health and disease causation (40, 41). Studies have investigated the adult microbiota in relation to its role in development of obesity and type 2 diabetes (42, 43), but there is limited information about the microbiota in childhood obesity. Ley et al. investigated the relationship between gut microbiota and obesity in adults and found decreased *Bacteroidetes* and increased *Firmicutes* in the obese adults compared to lean controls prior to dietary restriction. Body weight loss of 6% correlated with an increase in *Bacteroidetes* when participants were on a fat or carbohydrate restricted diet (42). Separate metagenome-wide association concluded that patients with type 2 diabetes had a moderate degree of gut microbial dysbiosis and increased numbers of opportunistic pathogens (43). A recent study has demonstrated that there is a difference in the microbiota of normal weight children compared to those who are overweight with an increase in *Firmicutes* and a decrease in *Bacteroidetes*, but did not assess NNS consumption (44).

Rodent models have demonstrated that the use of NNS such as saccharin, sucralose and aspartame led to alterations in the gut microbiota composition (18, 35–37). Abou-Donia et al. exposed male Sprague-Dawley rats to sucralose for 12 weeks and detected increased fecal pH and an overall reduction in beneficial fecal microbiota including *Bifidobacteriacea* (35). Mice exposed to aspartame and high fat diet had an increase in *Enterobacteriaceae* and *Clostridium leptum*, higher fasting glucose, and altered insulin mediated glucose clearance. This suggests that NNS may lead to gut dysbiosis and may result in metabolic dysregulation (18). To demonstrate possible causality and NNS associated gut microbiota alteration, Suez and colleagues transplanted the microbiota from saccharin exposed mice to germ-free mice. The microbiota of the saccharin exposed group had increased *Bacteroides* and *Clostridiales* with a decrease in *Lactobacilli*. Transplantation of this microbiota to a germ-free mice induced glucose intolerance with higher oral glucose tolerance measurements, suggesting that NNS can alter the microbiota and lead to metabolic dysregulation (37). The outstanding question is what are the biological mechanisms that shift the microbiota and induce metabolic dysregulation?

Multiple animal studies have demonstrated that NNS exposure can have bacteriostatic effects and that lead to changes in the microbiota composition. *E coli* colonies are reduced in both solid media and liquid cultures when cultured with sucralose (38). Rebaudioside A, an active ingredient from Stevia extract, exerted bacteriostatic effect on *E coli* growth *in vitro* and led reduction in *Bifidobacteriaceae* and *Lactobacillus* in young mice (45). Rebaudioside A also has been shown to increase *Akkermansia* while decreasing *Bacteroides* (46). The microbiota of young mice fed a diet that incorporated sucralose had increased *Firmicutes* and reduced *Bacteroidetes* populations in the feces. NNS have been shown to inhibit the anaerobic fermentation of glucose used by the rat microbiota as an energy source (39). Taken together, these studies are strong evidence that consumption of NNS can lead to microbiotic dysbiosis and metabolic dysregulation.

NNS are digested by gut bacteria into metabolites that can exert metabolic effects (47, 48). Studies on the association between obesity and energy harvesting pathways have found a higher concentration of short chain fatty acids (SCFAs) such as butyrate, acetate, and propionate in the gastrointestinal tract of overweight adults with central obesity and hypertension (47). Animals exposed to NNS also demonstrate an increased concentration of fecal SCFAs associated with metabolic dysregulation (18, 37). Aspartame-exposed mice had elevated fecal propionate levels, elevated fasting blood glucose, and impaired insulin tolerance test (18). Propionate can be taken up in the liver *via* the portal vein to serve as substrates for gluconeogenesis, lipogenesis, or as precursors to signaling molecules (47). The metabolic changes induced by NNS suggest the possibility of an indirect pathway where microbiota-derived SCFAs shift host metabolism.

The current literature examining the effects of NNS and the human gut microbiota are limited and no studies have been conducted in the pediatric population. Suez and colleagues followed a cohort of 381 non-diabetic adults and found a positive correlation between NNS consumption and the *Enterobacteriaceae* family, *Deltaproteobacteria* class, and the *Actinobacteria* phylum (37). This study also focused on a smaller subgroup healthy adults who were naive to NNS and was exposed to saccharin for 1 week. The participants who developed glucose intolerance were classified as responders while those who had no change in glycemic response were classified as non-responders. The microbiome of responders clustered differently and had pronounced compositional shifts at the end of the study. Fecal samples from these responders were transplanted to germ-free mice that then developed significant glucose intolerance (37). In contrast, Frankenfeld et al. analyzed food records and fecal samples from 31 adults and found a differences in microbial diversity between NNS consumers and non-consumers (49). Less is known about how the microbiota is affected in children as there is no published reports that have examined changes in the microbiota over long term exposure to NNS from early infancy through adolescence. Clinical studies are needed to examine whether the changes of the gut microbiota and the effects of NNS found in animal studies is also seen in pediatric populations.

## NNS Exposure and Glucose Homeostasis

While NNS may alter the gut microbiota composition and exert a secondary effect on host metabolism, the interaction of NNS and the endocrine pancreas is likely direct through the activation of the sweet taste present on the cell membranes of pancreatic beta cells (47, 48, 50). From *in vitro* models, acute exposure of pancreatic beta cells to NNS led to increased insulin secretion in response to a glucose load (51, 52). MIN6 cells, a pancreatic beta cell line, increased insulin secretion under glucotoxic conditions when exposed to rebaudioside A in a dose dependent response. Another study showed rebaudioside A increased beta cell mass and neuronal pancreatic innervation (18). However, the chronic effects of NNS exposure on pancreatic dysregulation and understanding the biological mechanism are unknown.

Clinical studies that investigated the acute effects of NNS consumption on glucose homeostasis in adults reported conflicting conclusions. Pepino and colleagues compared the effects of acute sucralose ingestion or water prior to a glucose challenge in obese subjects who were naive to NNS exposure. The sucralose group had higher peak plasma glucose concentration, insulin secretion rate, and an incremental increase in total insulin AUC compared to water-consuming controls (53). This suggests that acute ingestion of NNS causes impairment of glucose tolerance. In contrast, Wu et al. randomized healthy adults to receive water, sucralose with AceK, sucralose only or AceK only prior to glucose challenge and found no difference in post-prandial blood glucose concentration, insulin levels, or GLP-1 secretion (54). In a different study, Temizkan et al. found that acute exposure to sucralose enhanced GLP-1 release and lowered blood glucose in healthy subjects (55). Longitudinal studies have found an increased risk of NNS consumption and Type 2 diabetes (56, 57). The discordant outcomes in these studies highlight that the mechanisms that mediate NNS effects on glucose homeostasis are unclear. The above studies investigated the acute effects of NNS but the larger question still looms: how does chronic consumption of NNS produce long term metabolic effects and health outcomes?

Clinical studies and limited *in vitro* studies suggest that the physiological response to acute exposure of NNS on the endocrine pancreas can cause hyperglycemia and stimulate insulin secretion but does not explain the biological mechanisms that are dysregulated when diabetes and metabolic syndrome develop during chronic exposure to NNS. Animal models have suggested that NNS modulates the sodium glucose co-transporter 1 (SGLT-1) expression leading to an upregulation and higher glucose reabsorption through the GI tract thereby challenging the maintenance of glucose homeostasis (58, 59). Adults who consume NNS over long duration are shown to gain weight and increase adiposity, thus contributing to obesity. In turn, obesity becomes a risk factor for insulin resistance. The presence of unresolved hyperglycemia and prolonged increased insulin secretion also contribute to worsening insulin resistance over time (60). The consumption of NNS imposes a cyclic stress for beta cells. The disruption of the cephalic response increases caloric intake, increased caloric intake leads to increased adiposity and insulin resistance, thus requiring increased insulin secretion from beta cells. Taken together, this vicious stress cycle could lead to beta cell exhaustion resulting in beta cell death, decreased insulin secretion, increased hyperglycemia, and phenotypic manifestation as Type 2 diabetes.

While studies that have sought to explain how NNS consumption in adults may hasten the progression to type 2 diabetes, we have no insight into the effects of NNS on children, who are in a developmentally sensitive period for programming cellular responses. A crossover trial found that higher post-prandial blood glucose values in preschool children age who received aspartame and higher blood glucose levels in school age children who received saccharin compared to sucrose (61). As NNS exposure occurs in early childhood, it is imperative to pursue prospective studies to determine whether chronic NNS consumption throughout childhood leads to an increased risk of developing diabetes and metabolic diseases. The field should identify the biological mechanisms of how the chronic exposure of NNS lead to pancreatic dysregulation as implicated by animal studies of glucose intolerance and a higher risk of developing T2DM in adolescents who consume NNS beverages.

## Discussion

The substitution of table sugar with NNS has been used to combat the growing pandemic of obesity. The acute and long-term effects of NNS may contribute negative metabolic outcomes, including weight gain and adiposity, found in observational studies in children and adults (see**Table 1**). Animal studies suggest that the possible mechanisms for these metabolic outcomes include modifying the cephalic phase response to sweet taste with caloric intake and alteration in reward pathways. Studies must be conducted in pediatric populations to understand how the physiological mechanisms that NNS promotes can change the reward behavior system and lead to increased consumption of sweet taste and high calorie foods.

**Table 1:** NNs studies-research study designs and outcomes.

Study	Subjects	Age at baseline	Duration of follow up	NNS Intervention	Outcomes measured	Main findings
Pediatric studies						
Berkey et al. (20)	16771 children	9-14 yr	2 yr	NNS soda, servings, FFQ	BMI	Positive association of NNS and BMI gain in boys but not girls
Blum et al. (21)	166 children	8-9 yr	2 yr	NNS soda, 24 hr diet recall	BMI z-score, weight	Positive association of NNS intake and BMI z-score change
De Ruyter et al. (28)	641 children	5-12 yr	18 months	NNS soda, 1 can per day, compare to sugar sweetened beverages (SSB)	BMI z-score, weight, height ratio, fat mass, sum of skinfolds, waist circumference, % body fat	Reduced weight gain and fat accumulation with NNs vs. SSB
Ebbling et al. (23)	244 overweight and obese adolescents	14-16 yr	2 years	NNS beverage compared to sugar sweetened beverage	Change in BMI, weight	Small Increse in BMI at 1 year
Forshee et al. (19)	3311 children and adolescent	6-19 yr		NNS soda and NNS juice, g/day, survey	BMI	Positive association between NNs consumption and BMI
Laverty et al. (22)	13170 children	7-11 yr		NNS, serving/week caregiver reporting	BMI, % body fat	Higher BMI and % body fat with daily NNS consumption
Ludwig et al. (5)	548 children	11.7 ± 0.8 yr	19 months	NNS soda, servings/day, FFQ	BMI, overweight or obesity	No association of baseline NNS intake and change in BMI or incident overweight/obesity
Williams et al. (29)	32 overweight girls	10-16 yr	12 weeks	Calorie restricted diet with NNS soda offered, 24 hr diet recall	BMI, weight, blood pressure	No difference between groups
Animal studies						
Swithers et al. (16)	Sprague-Dawley male rats		5 weeks	Saccharin-sweetend yogurt	Weight, adiposity, total energy intake	Saccharin consumption increased weight gain, higher adiposity and increased total energy intake
Abou-Donia et al. (35)	Sprague-Dawley male rats		12 weeks	Sucnalose	Gut microbiota, fasting blood glucose	Reduction in beneficial fecal microbiota (bifidobacteriacea), higher fasting glucose
Suez et al. (37)	C57BW5mice		11 weeks	Saccharin	Gut microbiota	Increased bacteroides and Clostridalies decreased Lactobacili
Palmnas et al. (18)	Sprague-Dawley Male rats		8 weeks	Chow diet or high fat diet with aspartame (5-7 mg/kg/d in drinking water)	Gut microbiota, body fat, total calorie intake, fasting blood glucose, insulin tolerance test	Rats exposed aspartame consumed fewer calories, less weight gain but elevated fasting blood glucose and impaired insulin tolerance test, increased Firmicutes Bacteroidetes ratio in aspartame group and elevated short chain fatty acid propionate.

Diet plays a key role in shifting the balance of the gut microbiota and NNS have been shown to promote the growth of microflora that resembles those with an obese phenotype. The higher concentration of SCFAs found in animal models exposed to NNS suggest they exert an indirect effect of regulating metabolism (18, 37). NNS activates sweet taste receptors and potential pancreatic beta cells to secrete higher amounts of insulin. However, the pathways by which long term NNS exposure initiates insulin resistance and the interconnection with the imbalance of the gut microbiota leading to metabolic dysregulation remains to be investigated.

With the alarming rates of childhood obesity and the increased awareness of the ill effects of sugar sweetened beverages, the use of NNS as a sugar alternative for children has gained popularity. Exposure may even occur in early uterine life when mothers are consuming these sweeteners during pregnancy. The long-term health implications of chronic NNS exposure, starting from infancy through adolescence and into adulthood, are poorly understood. Prospective studies are clearly needed in the pediatric population to understand the physiological mechanisms that lead to obesity and metabolic dysregulation, including their impact on pancreatic, neuronal, and microbiome physiology. Animal models are critical to test hypotheses and determine biological mechanisms that drive the findings in epidemiological studies. The conclusion from future prospective longitudinal studies and animal models can have long term impacts on whether NNS should be recommended for the consumption by the pediatric population, and whether the potential weigh gain benefits are an acceptable for negative metabolic outcomes during this critical developmental period. Finally, despite potential common pathways, we caution that NNS compounds need to studies individually and only nuanced studies will truly provide NNS specific safety and usage guidance.

## Author Contributions

BS and SG conceived, wrote, and edited this manuscript. All authors contributed to the article and approved the submitted version.

## Funding

BS is supported by the Pfeiffer Foundation. SG is supported by the Harvey Family Foundation, the Paul Lester Foundation, and the Saban Research Institute. None of the aforementioned entities had any input in the development of this manuscript.

## Conflict of Interest

The authors declare that the research was conducted in the absence of any commercial or financial relationships that could be construed as a potential conflict of interest.
